# Modelling Tumour Oxygenation, Reoxygenation and Implications on Treatment Outcome

**DOI:** 10.1155/2013/141087

**Published:** 2013-01-14

**Authors:** Iuliana Toma-Dasu, Alexandru Dasu

**Affiliations:** ^1^Medical Radiation Physics, Stockholm University and Karolinska Institutet, 171 76 Stockholm, Sweden; ^2^Department of Radiation Physics UHL, County Council of Östergötland, Radiation Physics and Department of Medical and Health Sciences, Faculty of Health Sciences, Linköping University, 581 83 Linköping, Sweden

## Abstract

Oxygenation is an important component of the tumour microenvironment, having a significant impact on the progression and management of cancer. Theoretical determination of tissue oxygenation through simulations of the oxygen transport process is a powerful tool to characterise the spatial distribution of oxygen on the microscopic scale and its dynamics and to study its impact on the response to radiation. Accurate modelling of tumour oxygenation must take into account important aspects that are specific to tumours, making the quantitative characterisation of oxygenation rather difficult. This paper aims to discuss the important aspects of modelling tumour oxygenation, reoxygenation, and implications for treatment.

## 1. Introduction

Oxygenation is an important component of the tumour microenvironment and has a significant impact on the progression and management of cancer. Hypoxia, or reduced oxygenation, has been associated with restrained proliferation, apoptosis, necrosis, angiogenesis, the development of an aggressive phenotype, and resistance to treatment [1]. These aspects make the quantification of tumour oxygenation and the study of its impact on treatment approaches highly relevant topics in oncology research. Consequently, several methods have been proposed for measuring tissue oxygenation, employing polarographic, fluorescent, radionuclide, or magnetic resonance techniques [2]. The results of such measurements have generally been used for qualitative correlations with treatment outcome [3–9]. However, many of these methods are invasive or suffer from sampling or resolution limitations and these aspects may reduce the significance of the results. From this perspective, theoretical determination of tissue oxygenation through simulations of the oxygen transport process might be a practical alternative to characterise the spatial distribution of oxygen on the microscopic scale and its dynamics and to study its impact on the response to radiation. This paper aims to discuss the important aspects of modelling tumour oxygenation, reoxygenation, and implications for treatment. 

## 2. Origins of Tumour Hypoxia

One of the most striking features of solid tumours in comparison to normal tissues is their architecture of vascular networks. In normal tissues, the vasculature has developed to provide adequate supply of oxygen to all of the cells, while in tumours, the vascular network is inadequate, almost chaotic and the quality of the blood flowing in it can be quite poor [10, 11]. This is the result of the generally high proliferation rates of tumour cells in comparison to normal cells that allow the tumours to quickly outgrow the existing blood and nutrient supply. As sustained angiogenesis is one of the hallmarks of cancer [12], oxygen supply is restored in tumours through the formation of new blood vessels. This is a relatively slow process [13] and newly formed vessels are also outgrown by the proliferating cells. Furthermore, newly formed vessels in tumours usually originate on the venous side of the vasculature [10]. Newly formed vessels in most solid tumours are often dilated, tortuous and have incomplete or missing endothelial lining resulting in an increased vascular permeability [14]. They are also unable to respond to local or systemic signals for vasodilatation or vasoconstriction [11]. In addition, tumour vasculature is not accompanied by a lymphatic system hampering the drainage of interstitial fluid and by-products of cell metabolism which can further influence vascular flow. Indeed, changes in interstitial pressure are thought to be responsible for the temporary cessation or resumption of flow through tumour microvessels. Further, the preference of tumour cells to glucose metabolism leading to the formation of lactic acid as a by-product leads to an acidification of the extracellular fluid in tumours [14]. All of these effects lead to a unique microenvironment in tumours characterised by poor supply and steep gradients of oxygen and other nutrients.

Depending on the underlying mechanisms and their duration, two main types of hypoxia have been identified in tumours, chronic and acute. Limitations in oxygen diffusion from the blood vessels into the tissue lead to the development of diffusion-limited or chronic hypoxia, first described by Thomlinson and Gray [15]. In contrast, local disturbances in vessel perfusion lead to the appearance of perfusion-limited or acute hypoxia, described by Brown [16] and verified experimentally by Chaplin et al. [17]. This has also been referred to as cyclic or fluctuating hypoxia due to the temporary character of the disturbances and the restoration of the perfusion through the affected vessels. Further subtypes of hypoxia have been identified depending on the mechanisms behind the development of hypoxic regions in tumours [18]. An important feature of tumour oxygenation is that it is not static and that several mechanisms determine its dynamics on timescales ranging from a few minutes for acute hypoxia to several days for chronic hypoxia. Accurate modelling of tumour oxygenation would have to take into account all of these aspects. However, their underlying complexity makes the study of oxygen transport to tumours quite difficult, especially when the aim is to accurately quantify the oxygen distribution.

## 3. Intravascular Oxygen Transport

In vertebrates, oxygen transport from the lungs to tissues takes place through the circulatory system. Most oxygen is transported bound to the heme groups of the haemoglobin (Hb) molecule in red blood cells (RBCs). The binding of the oxygen to haemoglobin takes places in 4 steps corresponding to the 4 heme groups of the metalloprotein, since binding one oxygen molecule determines a conformational change of the Hb molecule that influences its ability to further bind other molecules. From this perspective, the kinetics of the oxygen-haemoglobin reaction is described by the Adair equation [19] giving the equilibrium relationship between the saturation of the Hb molecule and the oxygen tension. However, the Adair equation is seldom used for the mathematical modelling of oxygen transport since it cannot be inverted analytically to give the oxygen tension as a function of the saturation of the haemoglobin. Instead, the simpler Hill equation [20] is used:

(1) $$\begin{array}{c} S=\frac{{\left(p/p_{50,\text{Hb}}\right)}^{n}}{1+{\left(p/p_{50,\text{Hb}}\right)}^{n}}, \end{array}$$

where *S* is the fractional haemoglobin saturation, *p* is the local partial pressure of oxygen (or *p*O_2_), *p*_50,Hb_, is the *p*O_2_ value at which the haemoglobin is 50% saturated, and *n* is the Hill parameter.

The sigmoid relationship described by the Adair and Hill equations is usually known as the oxyhaemoglobin dissociation curve or the oxygen-haemoglobin equilibrium curve. This curve is affected by a number of factors including temperature, pH, and CO_2_ concentration [21]. Given the unique microenvironment of tumours with a low pH, the oxygen-haemoglobin equilibrium may be achieved at different oxygen tensions in tumours compared to normal tissues and there may also be differences between various normal tissues. In light of these expected differences, caution is advised at the generalisation of parameters determined for a particular tissue or during the extrapolation of the results achieved using these parameters.

Besides the bound component, modelled oxygen transport would also have to account for the free and dissolved oxygen in the red blood cells and blood plasma. Unbound oxygen represents only 1.5% of the total oxygen content of the blood in normal tissues, provided Hb is completely saturated [21]. However, this fraction might be higher for tumours, where vessels often originate on the venous side of the vasculature containing desaturated blood. The association between anaemia and cancer would further decrease the relative importance of the bound component. 

Modelling of intravascular transport has to account for both the free and the Hb-bound oxygen, as well as for the transport of oxygen through the membrane of the red blood cells. Popel [21] provided a comprehensive review of the earlier theoretical work on the particulate nature of RBC-plasma transport of oxygen. More recent approaches, however, have incorporated RBC-plasma transport into intravascular transport models accounting for intracapillary O_2_ gradients and giving the average flux of oxygen through the capillary wall [22]. These models have mainly been used for studying the oxygen delivery to muscles and have found that blood-tissue transport encounters an effective intravascular resistance (IVR) that could be quantified as a mass transfer coefficient. This is influenced by the assumptions for intravascular transport, the shape of the RBC or vessel permeability. This suggests that care has to be employed when extrapolating normal tissue models to tumours, as the poor endothelial lining of tumour microvessels increases their permeability, while the acidic tumour microenvironment could influence the shape of the RBC as well as the Hb dissociation curve.

## 4. Tissue Transport Models

The first attempts to theoretically describe the oxygen transport into tissue date from the beginning of the 20th century [23, 24] and were based on systems with simple geometries for which an analytical solution could be obtained. One of these early models, the Krogh tissue cylinder model, has been particularly important as it has served as the foundation for many subsequent studies. Its main assumptions were the following.

- *p*O_2_ distribution in tissue has a cylindrical symmetry and axial diffusion is not significant;
- tissue *p*O_2_ at capillary wall equals capillary *p*O_2_ (no intravascular resistance);
- oxygen transport in tissue takes place through passive diffusion;
- tissue diffusivity is independent of spatial position;
- transport phenomena are steady state.

Many models derived from the Krogh model have focused on the study of oxygenation in normal tissues as reviewed by Popel [21] and Goldman [22]. However, the assumptions of the Krogh tissue cylinder model have also been used by Thomlinson and Gray [15] and by Tannock [25] to describe theoretically the spatial distribution of tumour hypoxia. Later developments saw the introduction of models with more realistic vascular geometries to reflect the complexity of tumour vasculature, using Green functions or numerical methods to describe the tissue oxygenation [2, 26–31].

A general expression for the reaction-diffusion equation describing the oxygen transport in homogeneous tissues is shown in

(2) $$\begin{array}{c} \frac{\partial p}{\partial t}=\nabla \cdot \left(D\nabla p\right)-\nabla \cdot \left(\overset{\to }{u}p\right)-q+s, \end{array}$$

where *p* is the local *p*O_2_, *D* is the diffusion coefficient, $\overset{\to }{u}$ is the flow velocity, *q* represents the local consumption, and *s* is a source term.

In (2), the term ∇·(*D*∇*p*) describes the movement of oxygen through diffusion and the term $\nabla \cdot \left(\overset{\to }{u}p\right)$ describes the movement through convection. The convection term is usually neglected as the convective currents in the interstitial compartment of the normal tissues are estimated to represent only about 0.5–1% of the plasma flow. This may, however, not be the case of tumours, where the convective compartment could reach up to 15% [14]. The impact of neglecting the convective compartment in modelling tumour oxygenation is yet to be studied.

The rate of oxygen consumption in tissues, *q*, is thought to follow the Michaelis-Menten kinetics (3) as originally proposed by Tang [32], although variations of the equation have also been considered [33]:

(3) $$\begin{array}{c} q=q_{\max}\frac{p}{p+p_{50,\text{MM}}}, \end{array}$$

where *q*_max⁡_ is the maximum consumption rate, *p* is the local *p*O_2_, and *p*_50,MM_ is a parameter describing the *p*O_2_ at which the consumption rate halves. Equation (3) predicts an almost constant consumption rate at high oxygen tensions that is in agreement with the zero-order kinetics assumed by some of the earlier models [23, 25, 28, 34]. The Michaelis-Menten kinetics has, however, the advantage of more accurately describing the consumption at low *p*O_2_ and has been used in recent models [2, 27, 29, 31, 35–38].

The source term, *s*, in (2) has been used in different ways in various models. Some models assume that the source term could be set to zero since cells do not produce oxygen [29, 37–40], while others implement a different approach where the oxygen supply from the capillaries is described as a distributed source throughout the tissue with localised spikes at the vessel positions [30, 40].

Most of the studies have focused on a steady state of the system where ∂*p*/∂*t* = 0. This is in agreement with the observations by Hill [24], that the nonstationary evolution of the system is very short. Changes of oxygenation patterns due to perfusion limitations have time scales of minutes [16], while the formation of new blood vessels through angiogenesis takes place over several days [13] and therefore the steady state solution would apply in most situations. However, there might be some cases where the time-dependent oxygen transport would have to be considered, such as the onset of acute hypoxia after the cessation of blood flow, its disappearance following the reperfusion of capillaries, or cases when the oxygen supply through tumour capillaries has variations for longer time periods.

Solving (2) to determine tissue oxygenation requires setting boundary conditions for the limits of the modelled domain and even in this case various approaches have been used. For the interface between capillaries and tissue, one could neglect the intravascular resistance and use a Dirichlet boundary condition assuming that the *p*O_2_ value at the capillary wall equals the capillary *p*O_2_ [23, 29]. Alternatively, one could use a Robin boundary condition, assuming that the oxygen flux across the interface, that is, the vessel wall, must be continuous [37, 39, 40]. Assuming cylindrical symmetry for a capillary with radius *R* and thickness *w*, the diffusive flux on the tissue side of the vessel wall could be written as

(4) $$\begin{array}{c} F=-D_{\text{wall}}\int_{0}^{2\pi }\frac{\partial p}{\partial r}rd\theta =\frac{D_{\text{wall}}}{R}\frac{p_{\text{cap}}-p}{\ln\left(1-w/R\right)}, \end{array}$$

where *D*_wall_ is the diffusivity in the wall and *p*_cap_ is the capillary *p*O_2_. Assuming that the wall thickness *w* is much smaller than the external radius *R*, (4) becomes

(5) $$\begin{array}{c} F=\frac{D_{\text{wall}}}{w}\left(p_{\text{cap}}-p\right)=P_{m}\left(p_{\text{cap}}-p\right), \end{array}$$

where *P*_*m*_ is the vessel permeability.

The expression in (5) could be integrated over the surface of the capillary to obtain the net rate of oxygen entering the tissue. Thus, the source term in (2) could be written for each point representing the capillary as

(6) $$\begin{array}{c} s=\frac{1}{V}\int_{0}^{2\pi }F\cdot RL\;d\theta =\frac{1}{V}S\cdot P_{m}\left(p_{\text{cap}}-p\right), \end{array}$$

where *V* is the capillary volume and *S* is its area. Explicitly writing the volume and area of a cylinder, (6) becomes

(7) $$\begin{array}{c} s=\frac{1}{\pi R^{2}L}2\pi RL\cdot P_{m}\left(p_{\text{cap}}-p\right)=\frac{2P_{m}}{R}\left(p_{\text{cap}}-p\right). \end{array}$$

As expected, different boundary conditions would lead to different solutions of the reaction-diffusion equation, especially in the regions close to the vessels. However, Skeldon et al. [40] showed that for vessels with very high permeability, the Robin boundary condition leads to similar results as the Dirichlet boundary condition. If this is the case of tumour capillaries with increased permeability, it could provide an advantage when solving (2), since Dirichlet boundary conditions are easier to program.

Similarly, different approaches may be used for the outer boundaries of the simulated domain. These include Neuman boundary conditions setting the flux through the outer boundaries to zero, imposing periodic boundary conditions or other approaches [23, 29, 37]. The choice of the border conditions for the outer boundaries could also influence the amount of calculated hypoxia in the tissue as shown by Secomb et al. [37] who discussed the advantages and disadvantages of these approaches.

## 5. Modelling Parameters

An important aspect for the theoretical modelling of tissue oxygenation is the choice of parameters in (1)–(7). Experimental determination of these parameters is rather challenging and in many cases it involves the assumption of a model relating a measured quantity to the quantity to be determined. This highlights the importance of using parameters in the limit of the models for which they have been determined.

The least debated parameter in the literature is the average diffusivity of oxygen into tissue, with most authors agreeing on *D* = 2 × 10^−5^ cm^2^ s^−1^ [25, 28]. This could be explained from the point of view of the passive transport of oxygen through tissue and the relative homogeneity of soft tissues in the body.

In contrast, a relatively large range of values have been proposed for the maximum consumption rate, *q*_max⁡_, in (3) [10, 41–43], predominantly explained by the variability of the metabolic characteristics of tissues. As early as the beginning of the 20th century, Hill [24] observed significant differences in the consumption rates in active versus inactive muscle, while more recently Kallinowski et al. [44] showed that oxygen consumption in tumour cells decreases as cell quiescence develops. These are key observations because oxygen consumption is an important determinant of tissue oxygenation as shown by Secomb et al. [35], who found that a relatively modest reduction of tissue consumption may abolish tissue hypoxia, while a considerable increase of capillary oxygen content would be required for the same purpose. These results illustrate the difficulties in selecting the relevant consumption parameter for modelling. Indeed, significant differences may appear between tumour types or individuals depending on their metabolic activity, and intratumour heterogeneity could also be expected. Further confounding factors when determining the tissue consumption rate may reside in the method used for the determination and the assumptions made with respect to the oxygen supply rate or the invasiveness of the method used. Nevertheless, a relevant value for the maximum oxygen consumption rate in tumours that has been used in many studies is 15 mmHg s^−1^ [15, 25, 29].

Another parameter that is needed for the Robin boundary conditions (5) or for the distributed source models (7) is the permeability of tumour capillaries. Most of the permeability values available are relevant for normal tissue capillaries and these values have been used in studies investigating the oxygenation of tumours [30, 39, 40]. However, it has to be borne in mind that tumour vasculature has poor endothelial lining resulting in increased vascular permeability [14] and that normal tissue values may lead to an overestimation of the intravascular resistance of tumour capillaries.

Simulations of tumour oxygenation also require vascular oxygenation for the boundary conditions. Given the venous origin of newly formed vessels in tumours [10], a common assumption is that vascular *p*O_2_ in tumours is rather low [15, 25, 29, 37] and this is in agreement with experimental determinations of vascular oxygenations [45, 46]. Some studies have assumed a uniform oxygenation of the vessels [29, 30, 37, 39], while others have considered distributions of values [2, 28, 31]. Comparisons between simulations with the two assumptions have shown microscale differences between the resulting distributions, but no significant differences in the global characterisation of tissue oxygenation [2].

The vascular geometry is another important factor that has to be taken into account when simulating tumour oxygenation. Given the complexity of tumour vasculature, three-dimensional (3D) data would be required for the full characterisation of tumour oxygenation, but such images are difficult to obtain with existing *in vivo* imaging modalities due to their limited spatial resolution. Skin flap window chambers could be a solution to provide 3D geometries [35], but the thickness of the tissue in this model is much smaller than the chamber diameter. Alternatively, two-dimensional (2D) sections of *ex vivo* tissue samples could be used for the characterisation of vasculature and oxygenation. This approach was originally used by Thomlinson and Gray [15] for comparing the appearance of necrotic regions in lung tumours with calculations of tumour oxygenation and also in more recent studies employing tissue sections as input for simulations [28, 39]. An alternative approach is to use generated 2D maps of capillaries, either reflecting measured distributions of intervascular distances [2, 29, 31] or randomly generated distributions [30, 38, 40]. The former approach should be preferred as it has been shown that a full description of tissue vasculature is required for an accurate characterisation of tissue oxygenation. Average intervascular distance or the related quantity mean vascular density giving the number of vessels per unit area is one determinant of tissue oxygenation, the other being the shape of the distribution of intravascular distances [29].

Oxygen transport has also been coupled with the transport of other substances. Kirkpatrick et al. [47] used a mathematical model of oxygen and glucose mass transport in tumours to study the influence of kinetic and physical factors on metabolism. Other models are concerned with the transport and metabolism of tracers used in advanced imaging methods capable of visualising tumour hypoxia [30, 39, 40].

## 6. Modelling the Dynamics of Hypoxia

The dynamics of tumour hypoxia is an important aspect that has to be taken into consideration for many modelling studies. Indeed, angiogenesis and fluctuations in the perfusion of the blood vessels supplying the tumours have the potential to alter the oxygenation pattern, even in the absence of a therapeutic action upon the tumour cell population. These changes could in principle be modelled by assuming different spatial distributions of blood vessels over time, as the impaired perfusion of some vessels considered responsible for acute hypoxia would be equivalent to a decrease of the effective vascular density, while the appearance of new vessels would increase it. In this context it is important to note that even if the resulting distributions from repeated simulations are similar, in the case of acute hypoxia, different cells will be in different hypoxic subcompartments at each modelled time point and this dynamic process needs to be reflected in the modelled radiosensitivity of the cells.

Besides fluctuations in perfusion that are responsible for the cycling variation of the acutely hypoxic compartment, there are other mechanisms that may lead to changes in oxygenation during the course of a treatment. Radiation and other cytotoxic agents may induce growth inhibition in the irradiated cells that would subsequently lead to a decreased oxygen consumption in the surviving tumour cells [44] and thus improved oxygenation for previously hypoxic cells [35, 48]. On a longer timescale, the removal of the doomed cells near the blood vessels damaged by the cytotoxic agents might also improve the oxygenation of cells situated further away.

Another important aspect related to the timescale of hypoxia is the radiosensitivity of the affected cells. It is rather well known that decreased oxygen availability could lead to increased radioresistance of the hypoxic cells. Less known, however, is the effect of nutrient starvation that often accompanies chronic hypoxia, but not acute hypoxia. Indeed, it has been suggested [49, 50] that the two types of hypoxia might have different radiosensitivities due to changes in the repair capacity of the chronically hypoxic cells starved of oxygen and nutrients [51–55]. Consequently, differentiating between the two types of hypoxia as well as the time-dependent variation of the oxygen and nutrient availability might be needed for studies aiming to quantify the impact of certain treatment approaches on tumour containing both types of hypoxic cells. In this case, simulations would have to account for the perfusion status of the blood vessels in the tumour at various time points during the treatment and the impact on cellular radiosensitivity [56].

## 7. Tumour Oxygenation and Treatment ****Modelling

Theoretical simulations of oxygen transport to tumours have played a key role in understanding many aspects of tumour microenvironment that could influence treatment outcome. For example, theoretical modelling has allowed the characterisation of the gradients that may appear around the blood vessels, the study of the impact of the temporary closure of some capillaries, the radiobiological distinction between the two hypoxic compartments, or the effectiveness of various measurements methods. Comparisons between the maximum diffusion distance of oxygen into tissue and size of the viable rims of tumour cells around stroma or individual blood vessels led to the first descriptions of the appearance of chronic hypoxia in tumours [15, 25]. Simulations have also shown that several factors influence the relationship between tumour vasculature and oxygenation, indicating that there is an equivocal relationship between tissue oxygenation and vascular oxygenation, mean intravascular distance, or the number of unperfused vessels. Thus, mean vascular density is an important determinant of tumour oxygenation, but the relationship is modulated by the shape of the distribution of intervascular distances, the vascular oxygen content, and the number of closed capillaries [2, 29, 31]. This explains the failure to find in experimental studies a direct correlation between tumour oxygenation and vascular density [57].

Calculating the expected response from cell populations with different oxygenation requires a cell survival model with parameters that could be modified according to the radiation sensitivity of each compartment. According to the linear quadratic LQ model [58, 59], cell survival in a fully oxic population after a single radiation dose *d* is given by

(8) $$\begin{array}{c} SF_{\text{ox}}=\exp\left(-\alpha d-\beta d^{2}\right), \end{array}$$

where *α* and *β* are model parameters for oxic conditions. The modification of radiosensitivity in hypoxia could be accounted for through the use of modifying factors (OMFs) that are dependent on the local oxygen tension and the duration of hypoxia as in

(9) $$\begin{array}{c} SF_{\text{hyp}}=\exp\left[-\frac{\alpha }{\text{OM}{\text{F}}_{\alpha }\left(p{\text{O}}_{2},t\right)}d-\frac{\beta }{\text{OM}{\text{F}}_{\beta }^{\text{2}}\left(p{\text{O}}_{2},t\right)}d^{2}\right]. \end{array}$$

A noteworthy expression for the oxygen modification factors has been proposed by Alper and Howard-Flanders [60]:

(10) $$\begin{array}{c} \text{OMF}\left(p{\text{O}}_{2}\right)=\text{OE}{\text{R}}_{\max}\frac{k+p{\text{O}}_{2}}{k+\text{OE}{\text{R}}_{\max}p{\text{O}}_{2}}, \end{array}$$

where OER_max⁡_ is the maximum protection achieved in the absence of oxygen and *k* is a reaction constant around 2.5–3 mmHg [61, 62].

Cell survival for populations of cells characterised by a distribution of oxygenations could be obtained as the weighted sum of cell survival for each compartment:

(11) $$\begin{array}{c} SF_{\text{distr}}\left(d\right)=\sum_{i}w_{i}SF_{\text{hyp},i}\left(d\right), \end{array}$$

where *w*_*i*_ is the relative weight of each oxygenation compartment.

For fractionated treatments delivering *n* fractions of size *d*, different equations may be employed depending on the interfraction dynamics of oxygenation in the assumed population. Thus, for populations with static oxygenations, the cell survival is given by

(12) $$\begin{array}{c} SF_{\text{distr}}^{\text{static}}\left(n,d\right)=\sum_{i}w_{i}{\left[SF_{\text{hyp},i}\left(d\right)\right]}^{n}, \end{array}$$

while for populations with full dynamics of the oxygenation, cell survival is given by

(13) $$\begin{array}{c} SF_{\text{distr}}^{\text{dynamic}}\left(n,d\right)={\left[\sum_{i}w_{i}SF_{\text{hyp},i}\left(d\right)\right]}^{n}. \end{array}$$

Cell survival for other frequencies of oxygenations could be obtained by combining (12) and (13).

Theoretical simulations using (9)–(13) have shown significant differences in the predicted response depending on the assumed oxygenation of the tissue and these results indicate that full distributions of *p*O_2_ values, not only the hypoxic fraction, are needed for modelling [25, 56, 63]. This is particularly important in light of the equivocal relationship between tumour oxygenation and the above-mentioned parameters.

Equally interesting have been the results of simulations taking into account the possible radiobiological differences between acute and chronic hypoxic cells. Experimental studies have shown that chronically hypoxic cells may have low or depleted energy reserves which would impair their repair mechanisms [52–55], in contrast with acutely hypoxic cells that are repair competent. Correlating these findings with calculated oxygen distributions in tumours has suggested that the added biochemical sensitisation of chronically hypoxic cells could provide an explanation for the success of radiotherapy, as the chemical radioresistance conferred by hypoxia in general would otherwise require unrealistically high doses to control tumours containing hypoxic cells [49, 50, 56]. This comes as a confirmation of studies showing an improvement of control in experimental systems in the presence of chronically hypoxic cells [64]. In contrast, the presence of acute hypoxia in tumours leads to a worsening of the response to radiation therapy [56, 65, 66]. However, while the fluctuating character of the perfusion disturbances in tumour capillaries does not lead to a full “washout” of the radioresistance of acutely hypoxic cells, the fluctuations are essential for the success of fractionated therapy. Indeed, treatment schedules employing too few fractions (5 or fewer) might not allow enough interfraction reoxygenation opportunities to change the sensitivity of the hypoxic cells in tumours which would in turn lead to poor outcome [67]. This could have significant implications for stereotactic radiotherapy where extremely hypofractionated schedules should be pursued with caution [68].

Theoretical simulations have also been used to study the effectiveness of polarographic electrodes used to characterise tissue oxygenation [65, 69–72] or imaging with hypoxia-specific tracers [30, 39, 73–75]. It has thus been shown that averaging characteristic both to polarographic methods and to imaging methods with finite spatial resolution as well as differences in sensitivity of the measurement probes could lead to measured oxygenations that would be quite different from the tissue oxygenation [70, 73, 76], that in turn would translate into different predictions regarding tissue responsiveness to radiation therapy. This highlights the importance of carefully extrapolating measurements of tissue oxygenation to quantitative assessments of the expected response from radiation therapy.

*In vivo* imaging of hypoxia with dedicated tracers has received considerable attention in recent years as it could be used to quantify the spatial distribution and the severity of hypoxic areas in tumours for individualising or adapting the treatment according to biological factors [67, 77, 78]. In this context it has been shown that the dynamic character of hypoxia observed in clinical and experimental studies [79–84] has important implications for radiation therapy and it needs to be taken into account for treatment simulations. Indeed, neglecting it for prescriptions of highly heterogeneous dose distributions could lead to mismatches between planned dose hotspots and radioresistant hypoxic domains in tumours that would lead to poor outcome [67]. Furthermore, the limited spatial resolution of imaging methods could lead to poor rendering of the small hypoxic regions [76] that would translate into an underestimation of their radiosensitivity. However, this might be less of a problem for rapid interfraction reoxygenation that might ensure the success of dose painting techniques that do not take into account the microscale heterogeneity of hypoxia [85].

Besides these direct applications, theoretical modelling has proven a valuable tool in supplementing experimental data and for understanding the effects of factors that could not be studied individually or experimentally, in spite of uncertainties regarding tissue geometry, vascular O_2_ distributions, and *in vivo* parameters such as oxygen consumption rate [21, 22, 40]. It could also be used for more complex models studying the interplay between tissue oxygenation and cell growth and the macroscopic dynamics of tumours [86–88].

Far from being exhaustive, this review illustrates the importance of simulating the many complex facets tumour oxygenation. Considerable amount of work remains to be performed, both in updating the models of oxygen transport into tissue according to new findings and validations regarding the involved mechanisms and parameters and in terms of new opportunities for simulating treatments. Therefore, the increased interest in light of ion therapy [89] with its included potential of reducing the hypoxic radioresistance opens up new directions for research in terms of studying the impact of hypoxia on radiation treatment. New models for taking into account the response of hypoxic cells to high LET radiation are being developed [90] and will be tested with the ultimate aim to counteract hypoxic radioresistance and achieve maximum clinical benefit for patients. 
